# Knowledge, attitudes, practice of people toward the COVID-19 pandemics, and its impact in Afghanistan

**DOI:** 10.3389/fpubh.2022.983197

**Published:** 2022-11-17

**Authors:** Khwaja Mir Islam Saeed, Narges Neyazi, Khushal Nabizada

**Affiliations:** ^1^Afghanistan National Public Health Institute, Kabul, Afghanistan; ^2^International Campus, School of Public Health, Tehran University of Medical Sciences, Tehran, Iran; ^3^Department of Health System Development, World Health Organization, Kabul, Afghanistan; ^4^Afghanistan Institute for Strategic Studies, Kabul, Afghanistan

**Keywords:** Afghanistan, KAP survey, COVID-19 pandemic, socio-economic effect, knowledge, practice, attitude

## Abstract

COVID-19 pandemic disrupted the social and economic norms. Knowledge, Attitude and Practices studies are used to address the information gap for further strategic decision making to control the pandemic. This study aimed to find the level of Knowledge, Awareness, Attitudes, and behavioral practices of the people of Afghanistan about the COVID-19 and its impact on health and socio-economic dimension of their routine lives. We used a cross-sectional method with two stage sampling design. Data analysis was performed using SPSS v.20. The survey focused on adults including men and women all over the country to represent the country, including the urban and rural areas. Most of the respondents are in the age group of 21–30 years (46.5%); 60.15% of the participants are married. Almost 75% of females and 84% of males were literate and most participants have a bachelor's degree (34%). More than 80% of participants knew they can prevent themselves from contacting COVID-19 through hand washing frequently with soap and water and wearing a mask. More than 80% of participants responded that they would go for a lab test for detection of the virus as well as COVID-19 vaccination if it is available. Almost 35% reported always wearing a mask to prevent COVID-19 transmission; more than half of participants always wash their hands, more than 60% of them do not touch their eyes, nose, and mouth frequently. Nearly 60% of participants indicated that their household had problems satisfying food needs partly during the COVID-19 pandemic. Nearly half of participants believed that the government was successful in applying lockdown measures and in awareness rising (56.8 and 69.8%). The study findings provide some useful insight about the KAP of communities in Afghanistan, which could assist policy makers in public health to design and implement interventions based on the information gaps reported.

## Introduction

Coronavirus disease 2019 (COVID-19) is an emerged respiratory disease which is high infectious. On January 30, 2020, the WHO announced the outbreak as the Public Health Emergency of International Concern (PHEIC) (1), which later in March 2020, it was propagated as a pandemic. From the start of pandemic, five variants of concern (VOCs) of COVID-19 have been recognized in the world (Alpha, Beta, Gamma, Delta, and Omicron) (2). As of June 5, 2022, over 529 million confirmed cases and more than six million deaths globally have been reported. Based on WHO COVID-19 status report, only Alpha and Delta variants of COVID-19 were reported as of 26 June 2022 in Afghanistan (3) with a cumulative 182,352 cases and 7,722 deaths reported on 30 June 2022 (4).

Like other pandemics in the history, this pandemic also resulted in interruption of social and economic norms. Many studies were conducted to find out about these disruptions in the world (5–8). These studies assessed the level of Knowledge, Attitudes, Perceptions of selected populations and how the economy of people affected by the pandemic. To address the information gap for strategic decision making, a series of KAP studies were planned and implemented in Afghanistan too (9–11). Afghanistan, with a fragile health system, low economy and continuing conflicts has been more vulnerable to the pandemics. While the insufficient technical expertise and lab capacity to diagnose and treat the COVID-19 cases toughen up the severe effect of pandemic, health authorities declared that the actual number of positive cases could be higher than the numbers reported officially in the country (12). To control the pandemic, partial movement restrictions followed by bans on mass gathering and school closing were implemented in the country (13) which negatively affected the economic situation and worsened the poverty level in the country.

The level of Knowledge, Attitudes, and Practices (KAP) towards the disease affect the community practices and compliances with preventive measures. Having information on community perspectives regarding COVID-19 is crucial and will contribute to informed decision making, policy development and revision of risk communication and community engagement strategy by health authorities to fight against current and future pandemics.

In this study, we aimed to find the level of Knowledge, Awareness, Attitudes, and behavioral practices of the people in Afghanistan about COVID-19 and its impact on health and socio-economic dimension of their routine lives.

## Materials and methods

A cross-sectional study using a two-stage sampling design was conducted to provide information for addressing the overall purpose and specific objectives of the survey. The survey focused on adults (over the age of 18) including men and women all over the country to represent the country, including the urban and rural areas. The data was collected at community level by randomly approaching the households and interviewing the head of the family. All 34 provinces were included in the survey and data collection was completed within 3 months from February to April 2021 in the field. Persons who did not have consent and pregnant women were excluded from the study. Trained surveyors from the Afghanistan Institute of Strategic Studies (AISS) were given an orientation on how to collect data using a face-to-face structured questionnaire.

### Sample size and sampling strategy

For sample size calculations, key factors such as acceptable margin of error, desired level of confidence of the survey results, estimated baseline levels of the indicators, design effect of the sampling methodology, and anticipated non-response rate, were taken into consideration. The sample size of the KAP survey was calculated by considering a 5% margin of error, 95% confidence level, and 35% of the reference indicator. Due to cluster sampling, the design effect was assumed to 1.5. So, if we multiply 350 by 1.5, it will be 525. A response rate of 90% was assumed to participate. For the anticipated response rate of 90%, the current sample come to (525 ^*^1.11) 583 in each region. As the survey was planned to represent regional and national data, the sample was multiplied by five and finally the total sample size came to 2,915 households in 34 provinces. The sample size for each province was calculated based on its proportion to size. There was a two-stage cluster design; in the first stage, five districts were selected randomly by simple random sampling methods; in the second stage two areas were selected within each district.

### Variables and data collection

The research team developed a questionnaire containing seven sections: (1) general information, (2) Knowledge, (3) Attitude, (4) Practices, (5) socio-economic impact of COVID-19 on people lives, (6) the channels through which people receive information on COVID-19 and (7) the people's satisfaction on the government's response to COVID-19 pandemic. Most of the questions were designed based on a Likert scale to be answered. The answer options were selected based on the literature review and extracted from similar studies. The data collectors obtained the consent orally from the participants.

Data was collected using a face-to-face structured questionnaire by trained interviewers in the target areas. The team were consisting of two male and female interviewers supervised and monitored by a group of staff in Kabul and other provinces. After briefing the survey team, the pilot testing was conducted in the field and the tool was tested and improved. The study population consisted of a men and women in age group of 18 years and older who agreed to be interviewed in the study. However, severely ill, and pregnant women who are not able to be interviewed were excluded. The AISS team regularly supervised the process of the survey including recruitment of staff, training, and fieldwork to avoid any deviation. The questionnaires were distributed to participants who were literate and for those who were illiterate, the questions and answer options were read loudly by interviewers. The AISS team was responsible to timely execution of all activities including monitoring and quality assurance of field work. Each data collector entered the data into the excel sheet. A data entry clerk based in Kabul then cleaned, and edited the data, and entered them in an excel database.

### Data management and analysis

A data entry clerk based in Kabul conducted data quality check before analysis, and it was cleaned and validated in the Kabul AISS office. Data analysis was performed using SPSS v.20. Descriptive statistics were performed to calculate the proportions, rates, and ratios, which were used to prepare graphs and tables for better visualization of data. Frequencies of correct knowledge answers and various attitudes and practices were described. The statistical report is comprised of cross-tabulations of selected study variables representing knowledge (one question, have you heard about COVID-19/Coronavirus?), attitude (one question, if there is community transmission of COVID-19, will you participate in meetings, religious activities, events, and other social gatherings or any crowded place in areas?), and practices (two questions, do you wear mask to prevent and control COVID-19 transmission? And do you wash hands to prevent and control COVID-19 transmission?) as well as the demographic characteristics of respondents (residency place, gender, marital status, literacy, education, and income; Tables 1–**3**). We used Chi-Square to test bivariate relationships of study variables across the different background variables. Chi-Square test is used to test the relationship between a qualitative dependent variable and a qualitative independent variable. *P <* 0.05 was considered of statistical significance.

**Table 1:** Frequency distribution of the background characteristics of study participants (*N* = 2907).

**Categories**	**Male (%)**	**Female (%)**	**Total (%)**
Age in years			
20 and younger	167 (11.3)	303 (21.2)	470 (17.6)
21–30 Years	704 (47.7)	648 (45.3)	1352 (46.5)
31–40 Years	345 (23.4)	263 (18.4)	608 (20.9)
41–50 Years	148 (10.0)	142 (9.9)	290 (9.95)
51–60 Years	81 (5.5)	59 (4.1)	140 (4.8)
61 and older	31 (2.1)	16 (1.1)	47 (1.6)
Marital Status			
Single	485 (32.9)	588 (41.1)	1073 (37.0)
Married	986 (66.8)	766 (53.5)	1752 (60.15)
Widowed	4 (0.3)	73 (5.1)	77 (2.7)
Divorced/separated	1 (0.1)	4 (0.3)	5 (0.2)
Literacy			
Literate	1240 (84.0)	1075 (75.1)	2315 (79.55)
Illiterate	236 (16.0)	356 (24.9)	592 (20.45)
Education (system missing = 237 males and 355 females)			
No formal education	28 (2.3)	24 (2.2)	52 (2.25)
Primary	98 (7.9)	77 (7.2)	175 (7.55)
Secondary	129 (10.4)	129 (12.0)	258 (11.2)
High	316 (25.5)	242 (22.5)	558 (24)
Institutional diploma (14 pass)	158 (12.8)	231 (21.5)	389 (17.15)
Bachelor	459 (37.0)	334 (31.0)	793 (34)
Post-graduate	25 (2.0)	16 (1.5)	41 (1.75)
Madrassa	26 (2.1)	23 (2.1)	49 (2.1)
Language (missing = 7)			
Persian	878 (59.5)	951 (66.5)	1829 (63.0)
Pashto	488 (33.1)	368 (25.7)	856 (29.4)
Uzbek	94 (6.4)	99 (6.9)	193 (6.65)
Nooristani	12 (0.3)	10 (0.7)	22 (1.0)
Ethnicity			
Pashtun	511 (34.6)	416 (29.1)	927 (31.9)
Tajik	526 (35.6)	554 (38.7)	1080 (37.2)
Hazara	248 (16.8)	279 (19.5)	527 (18.1)
Uzbek	89 (6)	98 (6.8)	187 (6.4)
Aimaq	6 (0.4)	8 (0.6)	14 (0.5)
Baluch	13 (0.9)	11 (0.8)	24 (0.8)
Nooristani	7 (0.5)	8 (0.6)	15 (0.5)
Turkmen	13 (0.9)	12 (0.8)	25 (0.9)
Arab	21 (1.4)	10 (0.7)	31 (1.1)
Qezilbash	18 (1.2)	10 (0.7)	28 (1.0)
Sadat	24 (1.6)	25 (1.7)	49 (1.7)
Number of Family Member			
1–5	286 (19.4)	362 (25.3)	648 (22.3)
5–10	789 (53.5)	844 (59.0)	1633 (56.2)
10–15	280 (19.0)	176 (12.3)	456 (15.7)
15–20	69 (4.7)	34 (2.4)	103 (3.5)
20–25	24 (1.6)	8 (0.6)	32 (1.1)
25–30	14 (0.9)	2 (0.1)	16 (0.6)
Over 30	14 (0.9)	5 (0.3)	19 (0.7)
Home ownership			
Own	1032 (69.9)	792 (55.3)	1824 (62.7)
Rent	424 (28.7)	607 (42.4)	1031 (35.5)
Assurance	20 (1.4)	32 (2.2)	20 (1.4)
Occupation			
Day laborer	174 (11.8)	27 (1.9)	201 (6.9)
Student	136 (9.2)	255 (17.8)	391 (13.5)
Salaried employee (public)	281 (19.0)	194 (13.6)	475 (16.3)
Salaried employee	211 (14.3)	179 (12.5)	390 (13.4)
Farmer	47 (3.2)	9 (0.6)	56 (1.9)
Housewife	9 (0.6)	470 (32.8)	479 (16.5)
Small business	155 (10.5)	23 (1.6)	178 (6.1)
Self-employed	273 (18.5)	49 (3.4)	322 (11.1)
Jobless	190 (12.9)	225 (15.7)	415 (14.3)
Income (missing= 1) in AFN			
Less than 10,000	643 (43.6)	361 (25.2)	1004 (34.5)
10,000–19,999	378 (25.6)	137 (9.6)	515 (17.7)
20,000–49,999	89 (6.0)	54 (3.8)	143 (4.9)
50,000–100,000	24 (1.6)	14 (1.0)	38 (1.3)
More than 100,000	22 (1.5)	12 (0.8)	34 (1.2)
I donot have income at all	282 (19.1)	801 (56.0)	1083 (37.3)
Do not answer	37 (2.5)	52 (3.6)	(3.1)

### Ethical consideration

The study protocol was approved by Institutional Review Board (IRB) of Ministry of Public Health. The purpose of the survey was explained to all participants and informed consent from the interviewees was obtained. Each participant was informed that participation is voluntary, and they are free to withdraw of study at any time without any responsibilities. Participant confidentiality was maintained throughout the study. Data were entered into the database anonymously using the assigned identification numbers.

## Results

2907 respondents from 34 provinces of Afghanistan, with maximum rate (16.6%) from Kabul province and the minimum rate (0.6%) from Nimroz province completed the survey. The differences are due to population proportion living in these provinces. Almost 82% of participants in this study live in urban areas and the remaining (18%) live in rural areas. However, it should be noted that the study was mostly implemented in urban settings and due to existing insecurity, we had only 18% of data collection from rural areas.

**Figure 1:**
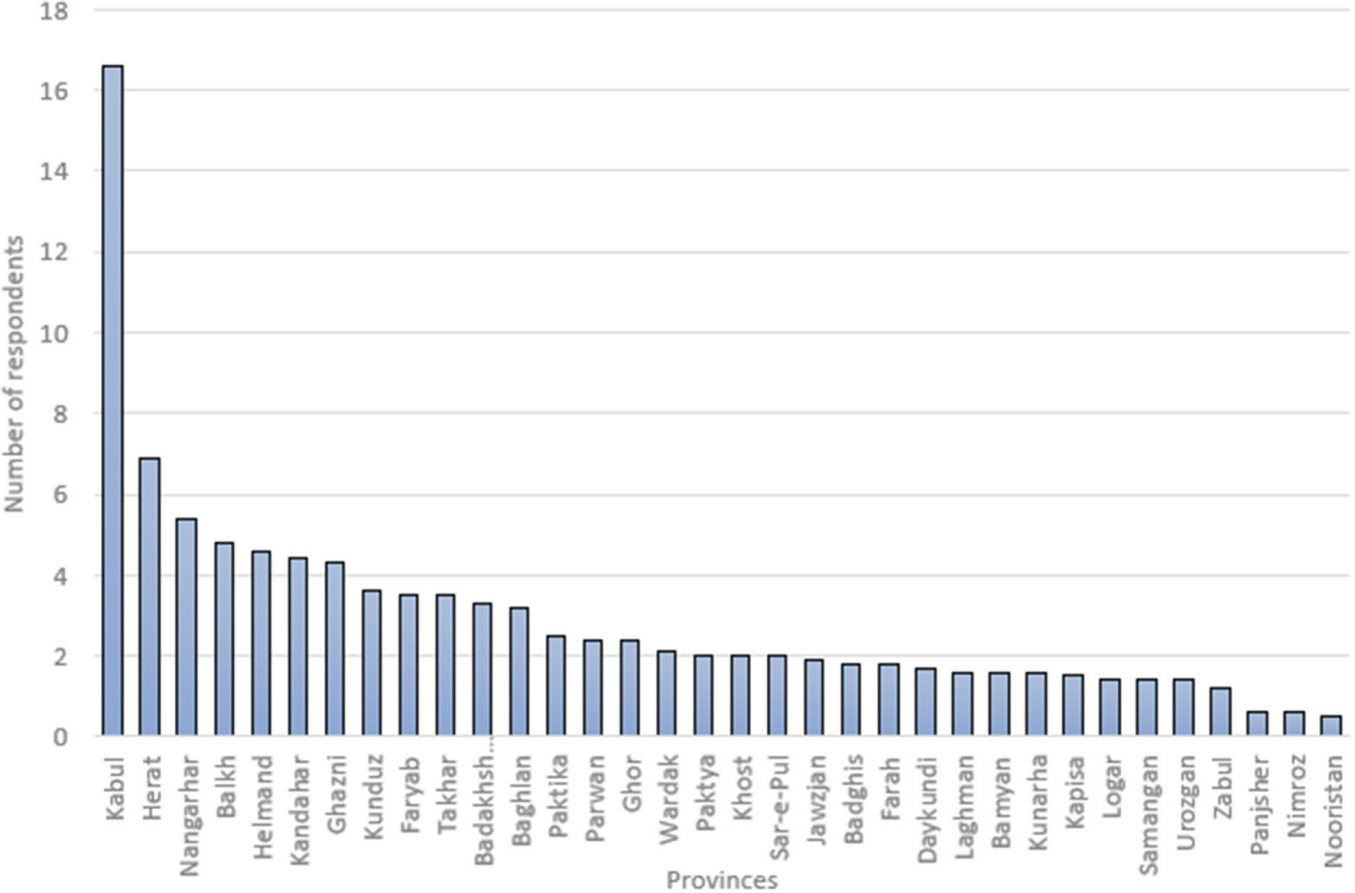
Proportion of respondents per province in KAP survey.

As shown in Figure 1, most participants were from regional provinces such as Kabul, Balkh, Nangarhar, Herat and Kandahar. The lowest participation was from, Nooristan, Panjshir and Nimroz provinces.

The socioeconomic and background characteristics of respondents are reflected in Table 1. As seen, most of the respondents are in the age group of 21–30 years (46.5%); 60.15% of the participants are married. Almost 75% of females and 84% of males were literate and almost one third of participants have a bachelor's degree (34%). Nearly 63% of participants speak in Dari, 29.4% speak in Pashto and the remaining speak in Uzbek, Nooristani and other languages. Most participants were Pashtun and Tajik (69.1%). Most of respondents owned their home (62.7%). One third of the female participants were housewives (32.8%); and the majority of the male participants were salaried employees (33.3%). More than half of the females did not have an income at all and nearly half of males had an income of less than 10,000 AFN (Afghani) monthly. It should be noted that the proportion of males (50.8%) and females (49.2%) were almost equal in this study. The highest number of family members were less than five people living under one ceiling.

### Knowledge about COVID-19

As reflected in Table 2, almost all the participants have heard about the Coronavirus. They believed that COVID-19 spreads via contact with droplets from an infected person, including cough (62.8%), going to crowded areas (59.6%), touching surfaces that someone infected has touched/cough on (49.5%), normal talking (43.3%), and by eating and drinking (38.4%) respectively. The subjects responded that fever (77.1%), cough (66.5%), headache (66.4%), sore throat (57.4%), and shortness of breath (57.7%) were the symptoms of COVID-19. More than three fourths of the participants believed that people who donot have the signs and symptoms of the COVID-19 can spread the disease. More than 80% of participants knew they can prevent themselves from contacting COVID-19 through hand washing frequently with soap and water and wearing a mask, and more than half of participants believed in the effectiveness of other measures such as avoiding close contact with those who are sneezing and coughing; avoiding handshakes, hugging, and kissing; avoiding going to crowded areas; and keeping physical distance. More than half of the respondents believed that suspected people with COVID-19 should be kept in quarantine for 2 weeks. Almost 41% of people who participated in this study believed that there (during period of this study) is an effective treatment available for COVID-19, and more than half (59.7%) believed that there is an effective vaccine available for this disease. Nearly 22% of participants did not know about any effective treatment and vaccine availability for COVID-19 during data collection. The participants responded that the elderly, people with hypertension, cardiovascular diseases, and suppressed immunity are the major populations who are at most risk for developing sever illness from COVID-19. A chi-square test of independence was performed to examine the relation between the demographic characteristics of participants and the level of their knowledge. As Table 3 shown, there is a significant relation between knowledge about COVID-19 and gender (*X* = 6.987, *p* = 0.008), marital status (*X* = 20.108, *p* = 0.000), literacy (***X*** **=** 34.317, *p* = 0.000), education (*X* = 19.227, *p* = 0.008), and income (*X* = 28.955, *p* = 0.002).

**Table 2:** Frequency distribution on knowledge of participant (*N* = 2907).

**Variable**	**Categories**	**Male (%)**	**Female (%)**	**Total (%)**
Have you heard about COVID-19/Coronavirus?				
	Yes	1466 (99.3)	1406(98.3)	2872 (98.8)
	No	10 (0.7)	25 (1.7)	35 (1.2)
How COVID-19 spreads? (The precents present Yes/Positive answers)				
	Normal talking	660 (44.7)	599 (41.9)	1259 (43.3)
	Food and water	566 (38.3)	910 (61.7)	1115 (38.4)
	Contact with droplets from an infected person, cough	949 (64.3)	877 (61.3)	1826 (62.8)
	Being in the same room with some who is infected	728 (49.3)	708 (49.5)	1436 (49.4)
	Close contact with someone infected	799 (54.1)	773 (54.0)	1572 (54.1)
	Touching surfaces that someone infected has touched/cough on	756 (51.2)	684 (47.8)	1440 (49.5)
	Going to crowded areas	893 (60.5)	841 (58.8)	1734 (59.6)
	Sharing the towels, toothbrushes	508 (34.4)	460 (32.1)	968 (33.3)
	Others	21 (1.4)	31 (2.2)	52 (1.8)
What are the symptoms of someone infected with COVID-19 (The precents present Yes/Positive answers)				
	Fever	1170 (79.3)	1072 (74.9)	2242 (77.1)
	Headache	966 (65.4)	963 (67.3)	1929 (66.4)
	Chills	643 (43.6)	666 (46.5)	1309 (45.0)
	Fatigue	594 (40.2)	578 (40.4)	1172 (40.3)
	Muscle ache (myalgia)	808 (54.7)	682 (47.7)	1490 (51.3)
	Sore throat	870 (58.9)	800 (55.9)	1670 (57.4)
	Cough	1056 (71.5)	876 (61.2)	1932 (66.5)
	Runny nose (rhinorrhea)	553 (37.5)	474 (33.1)	1027 (35.3)
	Shortness of breath (dyspnea)	839 (56.8)	838 (58.6)	1677 (57.7)
	Wheezing	280 (19.0)	269 (18.8)	549 (18.9)
	Chest pain	305 (20.7)	341 (23.8)	646 (22.2)
	Loss of smell or taste	502 (34.0)	541 (37.8)	1043 (35.9)
	Diarrhea	223 (15.1)	316 (22.1)	539 (18.5)
	Anorexia or loss of appetite	387 (26.2)	366 (25.6)	753 (25.9)
	I do not know	10 (1.3)	18 (1.3)	28 (1.0)
Do you think people who don't have the sign and symptoms of COVID-19 will spread the disease?				
	Yes	1045 (70.8)	995 (69.5)	2040 (70.2)
	Probably	264 (17.9)	261 (18.2)	525 (18.1)
	No	89 (6.0)	52 (3.6)	141 (4.9)
	I do not know	78 (5.3)	123 (8.6)	201 (6.9)
How can you prevent yourself from contacting COVID-19? (The precents present Yes/Positive answers)				
	Wash your hands frequently with soap and water	1264 (84.4)	1227 (85.7)	2473 (85.1)
	Wear mask	1208 (81.8)	1157 (80.9)	2365 (81.4)
	Avoid close contact with those who have sneezing and coughing	780 (52.8)	738 (51.6)	1518 (52.2)
	Avoid handshake, hug, and kissing	859 (58.2)	765 (53.5)	1624 (55.9)
	Avoid going crowded areas	831 (56.3)	745 (52.1)	1576 (54.2)
	Keep physical distance	684 (46.3)	592 (41.4)	1276 (43.9)
	I do not know	14 (0.9)	23 (1.6)	
How long the people suspected with COVID-19 should be kept in Quarantine? (missing = 2)				
	1 week	94 (6.4)	107 (7.5)	201 (6.9)
	2 weeks	770 (52.2)	783 (54.7)	1553 (53.4)
	3 weeks	468 (31.7)	407 (28.4)	875 (30.1)
	Other	57 (3.9)	57 (4.0)	114 (3.9)
	I do not know	85 (5.8)	77 (5.4)	162 (5.6)
Is there an effective treatment available for COVID-19 yet?				
	Yes	623 (42.2)	562 (39.3)	1185 (40.8)
	No	533 (36.1)	544 (38.0)	1077 (37.0)
	I do not know	320 (21.7)	325 (22.7)	645 (22.2)
Is there an effective vaccine available for COVID-19 yet?				
	Yes	898 (60.8)	838 (58.6)	1736 (59.7)
	No	251 (17.0)	250 (17.5)	501 (17.2)
	I do not know	327 (22.2)	343 (24.0)	670 (23.0)
Do you know which group of people with which problems are at most risk for developing severe illness of COVID-19? (The precents present Yes/Positive answers)				
	Hypertension	818 (55.4)	811 (54.9)	1734 (59.6)
	Cardiovascular	811 (54.9)	884 (61.80	1695 (58.3)
	Liver disease	479 (32.5)	501 (35.0)	980 (33.7)
	Renal disease	364 (24.7)	396 (27.7)	760 (26.1)
	Cancer	402 (27.2)	447 (31.2)	849 (29.2)
	Old age (elderly)	1109 (75.1)	916 (64.0)	2025 (69.7)
	Suppressed immunity	796 (53.9)	587 (41.0)	1383 (47.6)
	Chronic lung disease	355 (24.1)	301(21.0)	656 (22.6)
	Tuberculosis	312 (21.1)	340 (23.8)	652 (22.4)
	Pregnant women	271 (18.4)	403 (28.2)	674 (23.3)
	I do not know	32 (2.2)	32 (2.2)	(2.2)

**Table 3:** Knowledge score of COVID-19 by demographic characteristics.

**Characteristics**		**N**	**%**	***X***	***P*-value**
Residency place	Urban	2339	98.8	0.046	0.830
	Rural	533	98.9		
Gender	Male	1466	99.3	6.987	0.008
	Female	1406	98.3		
Marital Status	Single	1065	99.3	20.108	0.000
	Married	1730	98.7		
	Widowed	72	93.5		
	Divorced/Separated	5	100		
Literacy	Yes	2301	99.4	34.317	0.000
	No	571	96.5		
Education	No formal education	52	100	19.227	0.008
	Primary	170	97.1		
	Secondary	255	98.8		
	High	556	99.6		
	Institutional Diploma (14 pass)	387	99.5		
	Bachelor	791	99.7		
	Post-graduate	41	100		
	Madrassa	49	100		
Income	Less than 10,000	992	98.8	28.955	0.002
	10,000–19,999	513	99.6		
	20,000–49,999	143	100		
	50,000–100,000	38	100		
	More than 100,000	33	97.1		
	I do not have income at all	1069	98.7		
	Do not answer	83	93.3		

### Attitudes about COVID-19

The attitude related responses of participants are summarized in Table 4. As reflected in this Table more than half of the participants believed that the risk and threat of COVID-19 disease is very high. Also, nearly half of the participants believed that they have not contracted COVID-19 yet. More than 80% of participants responded that they would go for a lab test for detection of the virus as well as COVID-19 vaccination if it is available. Almost 67% of participants reported that they will not go to meetings, religious activities, big events, other social gathering, or any crowded places if a community transmission of COVID-19 exists. Most respondents were neutral or disagreed with this idea that there is no COVID-19 virus, and it is just a story devised by profit seeking companies and individuals. But nearly half of the participants believed that COVID-19 is a clear indication of the Almighty Allah's anger on wrong doers or committing sins. The relation between the attitude of participants and their demographic characteristics also was examined using a chi-square test. As Table 5 shown, there is a relation only between the gender (*X* = 18,610, *p* = 0.000) and the attitude of the participants but not between the participant's attitudes and residence place, marital status, literacy, education and income. It means the attitude toward COVID-19 is different between two genders and females had more positive attitude toward COVID-19 in this study.

**Table 4:** Frequency distribution on attitudes of participants toward COVID-19 disease (*N* = 2907).

**Variable**	**Categories**	**Male (%)**	**Female (%)**	**Total (%)**
In your opinion, how serious is the risk and threat of COVID-19?				
	Very high	798 (54.1)	871 (60.9)	1669 (57.4)
	High	380 (25.7)	331 (23.1)	711 (24.5)
	Moderate	212 (14.4)	163 (11.4)	375 (12.9)
	Low	33 (2.2)	20 (1.4)	53 (1.8)
	Very low	23 (1.6)	10 (0.7)	33 (1.1)
	I do not know	30 (2.0)	36 (2.5)	66 (2.3)
Taking into account the signs and symptoms, have you contracted COVID-19 yet? (missing = 93)				
	Yes	533 (36.1)	513 (35.8)	1046 (36.0)
	No	722 (48.9)	678 (47.4)	1400 (48.2)
	I do not know	176 (11.9)	192 (13.4)	368 (12.7)
Will you go for testing if a lab test for detection of the virus is available?				
	Yes	1237 (83.8)	1197 (83.6)	2434 (83.7)
	No	151 (10.2)	132 (9.2)	283 (9.7)
	I do not know	88 (6.0)	102 (7.1)	190 (6.5)
Will you vaccinate yourself, if COVID-19 vaccines are available?				
	Yes	1260(85.4)	1235 (86.3)	2495 (85.8)
	No	145 (9.8)	121 (8.5)	266 (9.2)
	I do not know	71 (4.8)	75 (5.2)	146 (5.0)
If there is community transmission of COVID-19, will you participate in meetings, religious activities, events, and other social gatherings or any crowded place in areas?				
	Yes	314 (21.3)	216 (15.1)	530 (18.2)
	No	938 (63.6)	1006 (70.3)	1944 (66.9)
	I do not know	224 (15.2)	209 (14.6)	433 (14.9)
Someone says, there is no COVID-19 virus, it is a story devised by profit seeking companies and individuals, what is your opinion?				
	Strongly agree	84 (5.7)	60 (4.2)	144 (5.0)
	Agree	165 (11.2)	120 (8.4)	285 (9.8)
	Neutral	360 (24.4)	454 (31.7)	814 (28.0)
	Disagree	546 (37.0)	488 (34.1)	1034 (35.6)
	Strongly disagree	321 (21.7)	309 (21.6)	630 (21.7)
Someone says, the COVID-19 is a clear indication of the Almighty Allah's anger on wrong doers or doing sins, what is your opinion?				
	Strongly agree	377 (25.5)	226 (15.8)	603 (20.7)
	Agree	432 (29.3)	353 (24.7)	785 (27.0)
	Neutral	309 (20.9)	443 (31.0)	752 (25.9)
	Disagree	214 (14.5)	235 (16.4)	449 (15.4)
	Strongly disagree	144 (9.8)	174 (12.2)	(10.9)

**Table 5:** Attitude score of COVID-19 by demographic characteristics.

**Characteristics**		**N**	**%**	***X***	***P*-value**
Residency place	Urban	1943	82.1	0.692	0.405
	Rural	434	80.5		
Gender	Male	1162	78.7	18.610	0.000
	Female	1215	84.9		
Marital Status	Single	857	79.9	5.727	0.126
	Married	1448	82.6		
	Widowed	68	88.3		
	Divorced/Separated	4	80		
Literacy	Yes	492	83.1	0.895	0.344
	No	2377	81.8		
Education	No formal education	42	80.8	1.661	0.976
	Primary	139	79.4		
	Secondary	209	81.0		
	High	460	82.4		
	Institutional Diploma (14 pass)	311	79.9		
	Bachelor	650	82.0		
	Post-graduate	34	82.9		
	Madrassa	40	81.6		
Income	Less than 10,000	802	79.9	8.511	0.203
	10,000–19,999	425	82.5		
	20,000–49,999	113	79.0		
	50,000–100,000	31	81.6		
	More than 100,000	25	73.5		
	I do not have income at all	909	83.9		
	Do not answer	71	79.8		

### Practice toward COVID-19

According to the results of this study almost 35% reported always wearing a mask to prevent COVID-19 transmission; more than half of participants always wash their hands, more than 60% of them do not touch their eyes, nose, and mouth frequently. The majority kept the physical/social distancing by remaining 2 m away from each other's, nearly half of participants cover their nose and mouth during coughing or sneezing with the elbow or a tissue and then throw the tissue away, and the majority often listen to and follow the directions of their health authorities. Most of the participants said that they or their family will call a doctor or go to a doctor's office or a hospital if they have been infected or if they have some of the common COVID-19's symptoms (84.6%), but most of the respondents prefer and trust governmental health facilities (60.5%). Meanwhile, in the case of a COVID-19 positive test, more than half of participants will stay home and self-isolate. In addition, nearly 40% of participants said that they will rarely or never participate in communal prayers, funerals, and celebrations such as Eid, or go to friend's houses or to work during the COVID-19 pandemic (Table 6). We chose two main indicators (wearing mask and washing hands) to find out the relation between the practice and demographic characteristics. As Table 7 shows, the practice of wearing mask has relation with residency place (people living in urban wear mask more than rural people), gender (female used mask more than male), marital status, literacy rate (literate people used mask more than illiterate people), and education level. As shown, the income level has no relation with using mask in this study. Also, our study shows that washing hands has relation with residency place (people in urban area wash their hands more), gender, marital status, education, and income level.

**Table 6:** Frequency distribution on practices of participants toward COVID-19 disease (*N* = 2907).

**Variable**	**Categories**	**Male (%)**	**Female (%)**	**Total (%)**
Do you wear mask to prevent and control COVID-19 transmission?				
	Always	388 (26.3)	616 (43.0)	1004 (34.5)
	Often	450 (30.5)	388 (27.1)	838 (28.8)
	Sometimes	401 (27.2)	244 (17.1)	645 (22.2)
	Rarely	155 (10.5)	126 (8.8)	281 (9.7)
	Never	82 (5.6)	57 (4.0)	139 (4.8)
Do you wash your hands to prevent and control COVID-19 transmission?				
	Always	656 (44.4)	933 (65.2)	1589 (54.7)
	Often	465 (31.5)	332 (23.2)	797 (27.4)
	Sometimes	260 (17.6)	119 (8.3)	379 (13.0)
	Rarely	74 (5.0)	35 (2.4)	109 (3.7)
	Never	21 (1.4)	12 (0.8)	33 (1.1)
Do you prevent touching your eyes, nose, and mouth frequently to prevent infecting with COVID-19?				
	Always	415 (28.1)	645 (45.1)	1060 (36.5)
	Often	508 (34.4)	428 (29.9)	936 (32.2)
	Sometimes	268 (18.2)	215 (15.0)	483 (16.6)
	Rarely	198 (13.4)	104 (7.3)	302 (10.4)
	Never	87 (5.9)	39 (2.7)	126 (4.3)
Do you practice physical/social distancing by remaining two meters away from others?				
	Always	256 (17.3)	418 (29.2)	674 (23.2)
	Often	423 (28.7)	363 (25.4)	786 (27.0)
	Sometimes	340 (23.0)	322 (22.5)	662 (22.8)
	Rarely	302 (20.5)	228 (15.9)	530 (18.2)
	Never	155 (10.5)	100 (7.0)	255 (8.8)
Do you cover your nose and mouth during coughing or sneezing with the elbow or a tissue and then throw the tissue away?				
	Always	551 (37.3)	776 (54.2)	1327 (45.6)
	Often	422 (28.6)	323 (22.6)	745 (25.6)
	Sometimes	273 (18.5)	198 (13.8)	471 (16.2)
	Rarely	185 (12.5)	109 (7.6)	294 (10.1)
	Never	45 (3.0)	25 (1.7)	70 (2.4)
Do you listen and follow the directions of your health authorities?				
	Always	569 (38.6)	698 (48.8)	1267 (43.6)
	Often	450 (30.5)	398 (27.8)	848 (29.2)
	Sometimes	300 (20.3)	230 (16.1)	530 (18.2)
	Rarely	119 (8.1)	94 (6.6)	213 (7.3)
	Never	38 (2.6)	11 (0.8)	49 (1.7)
What will you do if you and/or your family have been infected with COVID-19 or would have some of the common symptoms of COVID-19 such as dry cough, fever, and shortness of breath?				
	Do nothing/continue life as normal	63 (4.3)	82 (5.7)	145 (5.0)
	Call a doctor/medical professional	462 (31.3)	502 (35.1)	964 (33.2)
	Go to doctor's office/clinic	413 (28.0)	323 (22.6)	736 (25.3)
	Go to hospital	408 (27.6)	352 (24.6)	760 (26.1)
	Eating and drinking well	87 (5.9)	131 (9.2)	218 (7.5)
	I do not know	26 (1.8)	32 (2.2)	58 (2.0)
	No answer	17 (1.2)	9 (0.6)	26 (0.9)
What option would you prefer or trust if you and/or your family have been infected with COVID-19 and require medical treatment?				
	Going to governmental health facilities	929 (62.9)	829 (57.9)	1758 (60.5)
	Going to private health facilities	279 (18.9)	292 (20.4)	571 (19.6)
	Going to traditional health providers	76 (5.1)	67 (4.7)	143 (4.9)
	Using traditional treatment at home	192 (13.0)	243 (17.0)	435 (15.0)
Do you stay home and self-isolate when you tested positive or experience common COVID-19 symptoms?				
	Always	792 (53.7)	825 (57.7)	1617 (55.6)
	Often	329 (22.3)	268 (18.7)	597 (20.5)
	Sometimes	196 (13.3)	150 (10.5)	346 (11.9)
	Rarely	107 (7.2)	142 (9.9)	249 (8.6)
	Never	52 (3.5)	46 (3.2)	98 (3.4)
Have you participated in communal prayers, funerals including celebrating Eid and going to friend's houses and did not stop going to work during COVID-19 pandemic?				
	Always	227 (15.4)	309 (21.6)	536 (18.4)
	Often	311 (21.1)	240 (16.8)	551 (19.0)
	Sometimes	340 (23.0)	191 (13.3)	531 (18.3)
	Rarely	288 (19.5)	347 (24.2)	635 (21.8)
	Never	310 (21.0)	344 (24.0)	(22.5)

**Table 7:** Practice score of COVID-19 by demographic characteristics.

**Characteristics**		**Wearing Mask**			**Washing Hand**		
		**%**	***X***	***P*-value**	**%**	***X***	***P*-value**
Residency place	Urban	87.0	22.735	0.00	96.4	46.115	0.000
	Rural	79.0			89.4		
Gender	Male	83.9	6.280	0.012	93.6	15.536	0.000
	Female	87.2			96.7		
Marital Status	Single	90.4	52.955	0.000	96.5	23.903	0.000
	Married	83.5			94.7		
	Widowed	64.9			84.4		
	Divorced/Separated	80.0			100		
Literacy	Yes	89.2	125.357	0.000	96.3	33.485	0.000
	No	71.1			90.5		
Education	No formal education	78.8	53.244	0.000	92.3	20.519	0.005
	Primary	74.9			91.4		
	Secondary	87.2			96.1		
	High	90.1			95.7		
	Institutional Diploma (14 pass)	92.8			98.2		
	Bachelor	91.2			97.2		
	Post-graduate	90.2			95.1		
	Madrassa	89.8			95.9		
Income	Less than 10,000	85.0	13.646	0.34	92.6	23.295	0.001
	10,000–19,999	88.0			95.9		
	20,000–49,999	93.0			95.1		
	50,000–100,000	92.1			97.4		
	More than 100,000	82.4			97.1		
	I donot have income at all	83.7			96.7		
	Do not answer	86.5			98.9		

### Socio-economic impact of COVID-19

Nearly 60% of participants indicated that their household had problems satisfying food needs partly during the COVID-19 pandemic. Moreover, the majority (82.5%) stated that they applied for food rations during the lockdown. Most of the participants had to seek financial assistance and loans from their friends, relatives, colleagues, and communities (47.9%) during the lockdown. However, the majority also could afford to buy masks, disinfectants, and medicine when they were prescribed during COVID-19 pandemic. Almost 44% of participants stated that they lost part of their source of income in the mentioned period. This study shows that 45.3% of participant's families have students who faced problems in following their education during the pandemic period; the majority also believed that the COVID-19 pandemic caused an increase in the level of violence at family and/or in the community (Table 8).

**Table 8:** Frequency distribution on socio-economic Impact of COVID-19 pandemic (*N* = 2907).

**Variable**	**Categories**	**Male (%)**	**Female (%)**	**Total (%)**
During lockdown, did your household have problems satisfying its food needs?				
	Always	250 (16.9)	357 (24.9)	607 (20.9)
	Often	324 (22.0)	268 (18.7)	592 (20.4)
	Sometimes	364 (24.7)	303 (21.2)	667 (22.9)
	Rarely	267 (18.1)	288 (20.1)	555 (19.1)
	Never	271 (18.4)	215 (15.0)	486 (16.7)
Did you receive any food ration during lockdown?				
	Always	144 (9.8)	192 (13.4)	336 (11.6)
	Often	1248 (84.6)	1149 (80.3)	2397 (82.5)
	Sometimes	59 (4.0)	64 (4.5)	123 (4.2)
	Rarely	25 (1.7)	26 (1.8)	51 (1.8)
Have you sought financial assistance and loan from friends, relatives, colleagues, and community?				
	Always	71 (4.8)	160 (11.2)	231 (7.9)
	Often	248 (16.8)	234 (16.4)	428 (16.6)
	Sometimes	375 (25.4)	323 (22.6)	698 (24.0)
	Rarely	282 (19.1)	272 (19.0)	554 (19.1)
	Never	500 (33.9)	442 (30.9)	942 (32.4)
Could you afford buying mask and disinfections during the pandemic?				
	Always	247 (16.7)	279 (19.5)	526 (18.1)
	Often	293 (19.9)	291 (20.3)	584 (20.1)
	Sometimes	399 (27.0)	301 (21.0)	700 (24.1)
	Rarely	318 (21.5)	351 (24.5)	669 (23.0)
	Never	219 (14.8)	209 (14.6)	428 (14.7)
Could you afford buying medicine when you were prescribed during COVID-19 pandemic?				
	Always	212 (14.4)	228 (15.9)	440 (15.1)
	Often	249 (16.9)	305 (21.3)	554 (19.1)
	Sometimes	360 (24.4)	319 (22.3)	679 (23.4)
	Rarely	381 (25.8)	364 (25.4)	745 (25.6)
	Never	274 (18.6)	215 (15.0)	489 (16.8)
Have you lost your livelihood/sources of income during COVID-19 pandemic? (missing = 1086)				
	I totally lost my sources of income	417 (35.0)	191 (30.4)	608 (33.4)
	I lost part of my sources of income	548 (45.9)	260 (41.4)	808 (44.4)
	I did not lose my sources of income	228 (19.1)	177 (28.2)	405 (22.2)
Has any student in your family faced problems in following his/her education during pandemic?				
	Always	579 (39.2)	739 (51.6)	1318 (45.3)
	Often	486 (32.9)	306 (21.4)	792 (27.2)
	Sometimes	197 (13.3)	167 (11.7)	364 (12.5)
	Rarely	122 (8.3)	155 (10.8)	277 (9.5)
	Never	92 (6.2)	64 (4.5)	156 (5.4)
Has COVID-19 pandemic caused an increase in the level of violence at family and/or community?				
	Always	177 (12.0)	304 (21.2)	481 (16.5)
	Often	324 (22.0)	343 (24.0)	667 (22.9)
	Sometimes	352 (23.8)	277 (19.4)	629 (21.6)
	Rarely	300 (20.3)	267 (18.7)	567 (19.5)
	Never	323 (21.9)	240 (16.8)	(19.4)

### Source of information about COVID-19

Table 9 indicates the frequency distribution of the source of information of respondents about the COVID-19 pandemic. It reflects that 90.2% of participants have a mobile and more than half of them have access to internet sometimes. Most of the respondents also have radio and television in their home (60.0% and 82.9% respectively) and listen to the radio and watch TV often. The participants heard about COVID-19 mainly through television (67.8%); social media (42.3%); radio (35.1%); and health facility staff (32.7%). However, they mostly trust television (62.6%) and health facility staff (48.1%).

**Table 9:** Frequency distribution of source of information about COVID-19 (*N* = 2907).

**Variable**	**Categories**	**Male (%)**	**Female (%)**	**Total (%)**
Do you have mobile				
	Yes	1406 (95.3)	1216 (85.0)	2622 (90.2)
	No	70 (4.7)	215 (15.0)	285 (9.8)
Do you have access to internet?				
	Always	370 (25.1)	320 (22.4)	690 (23.7)
	Often	305 (20.7)	221 (15.4)	526 (18.1)
	Sometimes	281 (19.0)	197 (13.8)	478 (16.4)
	Rarely	174 (11.8)	176 (12.3)	350 (12.0)
	Never	346 (23.4)	517 (36.1)	863 (29.7)
Do you have radio at your home?				
	Yes	942 (63.8)	803 (56.1)	1745 (60.0)
	No	534 (36.2)	628 (43.9)	1162 (40.0)
Do you have a television at your home?				
	Yes	1183 (80.1)	1226 (85.7)	2409 (82.9)
	No	293 (19.9)	205 (14.3)	498 (17.1)
How often do you listen to the radio?				
	Always	202 (13.7)	225 (15.7)	427 (14.7)
	Often	250 (16.9)	197 (13.8)	447 (15.4)
	Sometimes	394 (26.7)	282 (19.7)	676 (23.3)
	Rarely	237 (16.1)	218 (15.2)	455 (15.7)
	Never	393 (26.6)	509 (35.6)	902 (31.0)
How often do you watch television?				
	Always	471 (31.9)	519 (36.3)	990 (34.1)
	Often	309 (20.9)	314 (21.9)	623 (21.4)
	Sometimes	325 (22.0)	289 (20.2)	614 (21.1)
	Rarely	148 (10.0)	125 (8.7)	273 (9.4)
	Never	223 (15.1)	184 (12.9)	407 (14.0)
Do you use social media?				
	Always	323 (21.9)	319 (22.3)	642 (22.1)
	Often	299 (20.3)	267 (18.7)	566 (19.5)
	Sometimes	333 (22.6)	218 (15.2)	551 (19.0)
	Rarely	210 (14.2)	276 (19.3)	486 (16.7)
	Never	311 (21.1)	351 (24.5)	662 (22.8)
How you heard about COVID-19?				
	Health facility staff	480 (32.5)	470 (32.8)	950 (32.7)
	MoPH authorities	362 (24.5)	324 (22.6)	686 (23.6)
	Community leaders	329 (22.3)	275 (19.2)	604 (20.8)
	Family member	312 (21.1)	433 (30.3)	745 (25.6)
	Social media	703 (47.6)	526 (36.8)	1229 (42.3)
	Religious scholars	333 (22.6)	231 (16.1)	564 (19.4)
	Mobile SMS	338 (22.9)	250 (17.5)	588 (20.2)
	Friends	460 (31.2)	407 (28.4)	867 (29.8)
	Radio	600 (40.7)	420 (29.4)	1020 (35.1)
	Television	997 (67.5)	973 (68.0)	1970 (67.8)
	Printed materials	134 (9.1)	118 (8.2)	252 (8.7)
	Calling to center 116	24 (1.6)	26 (1.8)	50 (1.7)
What is your preferred or trusted source of information?				
	Health facility	686 (46.5)	713 (49.8)	1399 (48.1)
	Community leaders	344 (23.3)	331 (23.1)	675 (23.2)
	Social medical	401 (27.2)	301 (21.0)	845 (29.1)
	Religious scholars	524 (35.5)	321 (22.4)	845 (29.1)
	Mobile SMS	232 (24.1)	185 (12.9)	417 (14.3)
	Friends	355 (24.1)	269 (18.8)	624 (21.5)
	Radio	483 (32.7)	359 (25.1)	842 (29.0)
	Television	905 (61.3)	914 (63.9)	1819 (62.6)
	Printed materials	198 (13.4)	122 (8.5)	320 (11.0)
	Call center 166	55 (3.7)	54 (3.8)	109 (3.7)

### Satisfaction of government's performance in response to COVID-19

Nearly half of participants believed that the government was successful in applying lockdown measures and in awareness rising (56.8% and 69.8%) during the COVID-19 pandemic in Afghanistan. Analysis showed that 44.2% of respondents believed that the government was/is successful in providing isolation centers in the mentioned period. However, most of them were not satisfied in terms of the provision of treatment services (49.7%), referral/ambulance services (50.2%), death management services (51.3%), or the provision of food and essential needs to poor families by the government (67.8%) during the pandemic, respectively (Table 10).

**Table 10:** Level of satisfaction from government by participants related to COVID-19 control and prevention (*N* = 2907).

**Variable**	**Categories**	**Male (%)**	**Female (%)**	**Total (%)**
The government was/is successful in applying lockdown measures during COVID-19 pandemic in Afghanistan? (missing = 3)				
	Very satisfied	148 (10.0)	178 (12.4)	326 (11.2)
	Satisfied	564 (38.2)	616 (43.0)	1180 (40.6)
	Neutral or does not know about the services	180 (12.2)	205 (14.3)	385 (13.2)
	Dissatisfied	359 (24.3)	299 (20.9)	658 (22.6)
	Very dissatisfied	223 (15.1)	132 (9.2)	355 (12.2)
The government of Afghanistan was/is successful in awareness raising during COVID-19 pandemic?				
	Very satisfied	225 (15.2)	216 (15.1)	441 (15.2)
	Satisfied	797 (54.0)	790 (55.2)	1587 (54.6)
	Neutral or does not know about the services	95 (6.4)	142 (9.9)	237 (8.2)
	Dissatisfied	245 (16.6)	216 (915.1)	461 (15.9)
	Very dissatisfied	114 (7.7)	67 (4.7)	181 (6.2)
The government of Afghanistan was/is successful in providing isolation centers during COVID-19 pandemic?				
	Very satisfied	137 (9.3)	105 (7.3)	242 (8.3)
	Satisfied	530 (35.9)	515 (36.0)	1045 (35.9)
	Neutral or does not know about the services	209 (14.2)	299 (20.9)	508 (17.5)
	Dissatisfied	436 (29.5)	374 (26.1)	810 (27.9)
	Very dissatisfied	164 (11.1)	138 (9.6)	302 (10.4)
The government of Afghanistan was/is successful in providing treatment services during COVID-19 pandemic?				
	Very satisfied	73 (4.9)	71 (5.0)	144 (5.0)
	Satisfied	482 (32.7)	478 (33.4)	960 (33.0)
	Neutral or does not know about the services	126 (8.5)	231 (16.1)	357 (12.3)
	Dissatisfied	542 (36.7)	471 (32.9)	1013 (34.8)
	Very dissatisfied	253 (17.1)	180 (12.6)	433 (14.9)
The government of Afghanistan was/is successful in providing referral/ambulance services during COVID-19 pandemic?				
	Very satisfied	81 (5.5)	47 (3.3)	128 (4.4)
	Satisfied	376 (25.5)	314 (21.9)	690 (23.7)
	Neutral or does not know about the services	214 (14.5)	417 (29.1)	631 (21.7)
	Dissatisfied	528 (35.8)	413 (28.9)	941 (32.4)
	Very dissatisfied	277 (18.8)	240 (16.8)	517 (17.8)
The government of Afghanistan was/is successful in providing death management services of those died due to COVID-19 during the pandemic?				
	Very satisfied	55 (3.7)	39 (2.7)	94 (3.2)
	Satisfied	370 (25.1)	347 (24.2)	717 (24.7)
	Neutral or does not know about the services	244 (16.5)	362 (25.3)	606 (20.8)
	Dissatisfied	577 (39.1)	463 (32.4)	1040 (35.8)
	Very dissatisfied	230 (15.6)	220 (15.4)	450 (15.5)
The government of Afghanistan was/is successful in providing food and essential needs to poor families?				
	Very satisfied	46 (3.1)	45 (3.1)	91 (3.1)
	Satisfied	304 (20.6)	291 (20.3)	595 (20.5)
	Neutral or does not know about the services	92 (6.2)	158 (11.0)	250 (8.6)
	Dissatisfied	467 (31.6)	481 (33.6)	948 (32.6)
	Very dissatisfied	567 (38.4)	456 (31.9)	1023 (35.2)

## Discussion

Like other KAP surveys, this study was conducted to find the level of Knowledge, Attitudes, and Practices towards COVID-19, as well as the gaps and behavioral patterns among various subgroups in Afghan society. Using result of study effective public health interventions can be designed and implemented to improve the situation (14). This study was conducted almost one year after the detection and prevalence of the COVID-19 pandemic in Afghanistan; that is why the findings of this study reflected that the study participants had sufficient knowledge about COVID-19 all over the country. In addition, they knew adequately the modes of transmission of the virus through the respiratory droplets of infected people. Most respondents are literate with higher education which is certainly an important input for better knowledge and appropriate practices.

Furthermore, the respondents were able to correctly identify the signs and symptoms of the disease. This high level of knowledge about COVID-19 and its preventive measures shows that the campaigns and communications conducted by government have worked. Participants had knowledge and information about preventive measures such as hand washing, personal hygiene, cough hygiene, using a mask, and social distancing. These are similar with other studies' findings (15, 16) and 1 year of exposure to the pandemic could be the reason for this high level of knowledge on various aspects of the disease in the community. Although having knowledge directly affects the behavior of people, it is not easy to claim that a certain level of knowledge is sufficient to cause positive changes. However, it should be noted that the impact of knowledge on health behaviors has been investigated in various studies (17–19). Our study also showed that small portion of respondents believed on misconceptions caused by “infodemic”. WHO says “*an infodemic is an overabundance of information, both online, and off line. It includes deliberate attempts to disseminate wrong information to undermine the public health response and advance alternative agendas of groups or individuals. Mis- and disinformation can be harmful to people's physical and mental health; increase stigmatization; threaten precious health gains; and lead to poor observance of public health measures, thus reducing their effectiveness and endangering countries' ability to stop the pandemic*” (20). The infodemic has been a big challenge during the COVID-19 pandemic (21–23). Therefore, specific procedures should be followed to provide accurate information to neutralize myths and misguided information through the internet and social media. This is an important finding because during the implementation of this study, the vaccine was widely available and still effective treatment was not available for the disease. These are important points that should be focused on health education and promotion interventions.

In addition, most respondents believed that COVID-19 is a viral disease with some believing it could be Almighty Allah's anger on wrong doers or committers of sins. A similar expression was given by 60% of Muslim communities in Nigeria where it was perceived that the pandemic is due to God's punishment (24). This is an important point to be focused on by the government as well as religious leaders to discuss and develop interventions for betterment. Levels of knowledge about the source of infection, modes of transmission and ways of prevention are important findings in this study. Another important finding is the level of social distancing by respondents and listening to messages from health authorities. However, there is a big gap between having knowledge and the actual attitudes and practices of the community. Meaning that appropriate knowledge has not always influenced good attitude or practice. For instance, only one third of respondents reported wearing mask when needed and half of them wash their hands frequently. Two third are not touching their mouth, nose, and eyes. These are the areas for more focus in future sessions for risk communication.

The pandemic destroyed the socioeconomic status of the countries throughout the world. In Afghanistan, almost two third were negatively affected during mobility restriction last year limiting their ability to earn and provide food. The coping mechanisms such as applications of rations and seeking financial assistance and loans during lockdown were implemented by households. Almost half of participants stated that they had lost part of their source of income during the lockdown period. Furthermore, the prices of all goods and services were rising during restrictions/lockdowns, for sure it has affected the price of mask, sanitary material, and disinfectants. Such impact of pandemic has been recorded in other studies as well (25). The increased level of violence at home and the disturbance to the education of children are important points to be focused while implementing restriction of normal activities and other measures. As the majority had access to mobile, radio, TV and sometimes to internet facilities, the main sources of information were mostly radio and TV followed by social media and healthcare workers. However, the most trusted channels of information were television and healthcare staff. Therefore, the most high-ranking channels such as TV, radio and healthcare workers should be utilized for risk communication messages and community engagement. The government's level of success in response to this pandemic was assessed as satisfactory by half of respondents. The main reasons for failure of the health system were identified as poor treatment services, referral/ambulance services, death management services, or inadequate provision of food and essential needs to poor families. These are the main lessons to be learned for future waves of this pandemic as well as future emergencies. This study had few limitations which needs to be expressed. First, like all other studies, it was difficult to include and consider all related factors with respect to Knowledge, Attitudes, and Practices. In addition, although we designed the study to collect data from urban and rural setting, due to existing insecurity in rural areas, the study has been mostly implemented in urban settings (82% of the respondents) with good access to information and better socioeconomic status and the rural area, certainly with less access to key source of information as their counterparts, are missing. Therefore, the results would be generalizable nationally only to people living in urban setting.

## Conclusion

The study findings provide some useful insight about the KAP of communities in Afghanistan, which could assist policy makers in public health to design and implement interventions based on the information gaps reported. Considering the findings of this study, the following recommendations are given:

- All stakeholders in controlling the COVID-19 pandemic can use the information from this KAP study to revise their risk communication strategies to control COVID-19 efficiently The government and public health authorities should establish and implement appropriate policies and interventions that are tailored to the level of understanding of communities. According to findings of our study, males and people living in urban areas, has good knowledge. In addition, based on our findings, illiterate people do not use mask or wash their hands to prevent getting infected with COVID-19. So, the health authorities should focus mostly on females and the illiterate population living in rural settings with proper strategies to enhance their knowledge and promote their practice toward controlling and preventing COVID-19 in their community.
- We recommend for further KAP study among population living in rural setting to fill the gap presented in our study and generate evidence for people living in rural areas.
- Public health officials should further enhance the knowledge of communities living in rural areas while considering the contextual factors which adversely affect the transfer of knowledge to behavior change such as some cultural and traditional related factors.
- Information about modes of transmission of the virus should be communicated clearly targeting all misconceptions and rumors. The survivors from COVID-19 and fully vaccinated individuals with high-risk behaviors should be encouraged to share their experiences as a point for mobilizations.
- Despite having good knowledge and attitudes, the health authorities should focus more on awareness campaigns at the community level. There are a wide range of channels to be used such as face-to-face health education, posters, billboards, social channels, radio, and TV advertisements to fill these practice gaps and improve the situation.
- There is a need for more relevant communication, and engagement of communities by local and religious leaders in the promotion of adherence to preventive measures. Religious leaders especially Mullahs from mosques should be enlightened about various aspects of the pandemic and its negative impacts because their advice and recommendation are sufficiently working in a sensitive and religious communities like Afghanistan.
- Information and health education (IEC) programs and behavior change communication interventions (BCC) in health promotion department of MoPH with respect to COVID-19 are important to maintain appropriate knowledge and improve positive practices by targeting people with low knowledge and education levels.
- A good level of coordination should be made between various parties involved in fighting the COVID-19 pandemic in Afghanistan, especially those organizations who reach to poor people and provide them with in-kind and financial assistance. Different international and national donors such as UN agencies (World Food Program, World Health Organizaion, UNICEF, UNDP), provincial governments, provincial public health directorates and the Ministry of Public Health should be the main members of this coordination.
- As the majority proportion of Afghanistan's population is below the age of 18 and can be the source of transmission for the COVID-19 virus, it is recommended that another KAP survey be conducted among the 7–18-year-old age group population (especially school students) to measure their Knowledge, Attitude, and Practices toward the COVID-19 pandemic.
- Simultaneously with emergence of different types of COVID-19 in the world as well as Afghanistan, the main actors in combating with this pandemic, especially the MoPH and other international stakeholders such as WHO should update the public on the signs and symptoms of each variant and the ways of preventing and treating it.
- Recent power transfer and change of government in Afghanistan also hit the health system hardly, a situation analysis through the rapid assessments, along with re-planning for combatting COVID-19 is highly recommended.

## Data availability statement

The original contributions presented in the study are included in the article/supplementary files, further inquiries can be directed to the corresponding author.

## Author contributions

NN, KS, and KN designed the research and wrote the paper. NN analyzed the data and had primary responsibility for final content. All authors read and approved the final manuscript.

## Funding

The funding for implementation of this study was granted by Open Society Foundation.

## Conflict of interest

The authors declare that the research was conducted in the absence of any commercial or financial relationships that could be construed as a potential conflict of interest.

## Publisher's note

All claims expressed in this article are solely those of the authors and do not necessarily represent those of their affiliated organizations, or those of the publisher, the editors and the reviewers. Any product that may be evaluated in this article, or claim that may be made by its manufacturer, is not guaranteed or endorsed by the publisher.
